# Gochujang prepared using rice and wheat koji partially alleviates high‐fat diet‐induced obesity in rats

**DOI:** 10.1002/fsn3.1443

**Published:** 2020-02-06

**Authors:** Hee‐Kyoung Son, Hye‐Won Shin, Eun‐Seok Jang, Byoung‐Seok Moon, Choong Hwan Lee, Jae‐Joon Lee

**Affiliations:** ^1^ Department of Food and Nutrition Chosun University Gwangju Korea; ^2^ Foods Research Institute CJ CheilJedang Corp. Suwon Korea; ^3^ Department of Bioscience and Biotechnology Konkuk University Seoul Korea

**Keywords:** anti‐obesity, *Gochujang*, high‐fat diet, rice *koji*, secondary metabolite, wheat *koji*

## Abstract

The aim of this study was to compare whether *gochujang* products prepared using giant embryo rice *koji* (rice *gochujang*, RG) and wheat *koji* (wheat *gochujang*, WG) have anti‐obesity effects on rats fed a high‐fat diet (HFD), who served as a model for obesity. The nutritional composition of RG and WG including proximate constituents, amino acid and fatty acid compositions were investigated. Consequently, the secondary fermented metabolites were analyzed in RG and WG by ultrahigh‐performance liquid chromatography and mass spectrometry. Rats were fed a HFD containing 10% RG powder (HFD‐RG) or 10% WG powder (HFD‐WG) for 8 weeks. Body weight gain, weights of liver, epididymal, retroperitoneal, perirenal, and total white fat pads, and levels of serum triglyceride (TG), total cholesterol, low‐density lipoprotein cholesterol, and leptin were lower in all *gochujang* groups than in the HFD group. Furthermore, RG and WG treatment decreased the hepatic TG content and lipid accumulation and significantly reduced the size of epididymal adipocytes. These effects are probably mediated through inhibition of hepatic fatty acid synthase, acetyl CoA carboxylase, malic enzyme, and adipose tissue lipoprotein lipase activities. The anti‐obesity effect was slightly greater in the HFD‐RG group than in the HFD‐WG group. This effect may be attributed to secondary metabolites, such as capsaicin, genistein, daidzein, soyasaponin, and lysophosphatidylcholines, contained in *gochujang* prepared using giant embryo rice or wheat *koji*.

## Graphical abstract

The aim of this study was to compare whether gochujang products prepared using giant embryo rice koji (RG) and wheat koji (WG) have anti‐obesity effects on rats fed a high‐fat diet (HFD). Anti‐obesity effects were slightly greater in the HFD‐RG group than in the HFD‐WG group. These effects were attributed to secondary metabolites, such as capsaicin, genistein, daidzein, soyasaponin and lysophosphatidylcholines, contained in gochujang prepared using giant embryo rice or wheat koji.

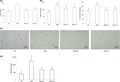

## 1 INTRODUCTION

*Gochujang* (fermented red pepper paste) is a traditional Korean soybean‐based fermented food; it is a seasoned food that plays a very important role in the diet of Koreans, along with soy sauce (*ganjang*), soybean paste (*doenjang*), and fast‐fermented soybean paste (*cheonggukjang*). It is prepared with fermented soybean by primarily adding grains and red pepper (*Capsicum annum* L). *Gochujang* has a unique flavor that combines the pungent flavor of the red pepper, sweet taste from the hydrolysis of starches, savory taste of proteolyzed amino acids and peptides from soy protein, salty taste, and sour taste of organic acids from microbial fermentation (Kang & Back, 2014; Oh, Kim, & Shin, 2006).

*Gochujang* possesses various biological properties, including anti‐atherosclerotic (Cha et al., 2013; Hyun, No, Lim, Cha, & Choi, 2007; Lim et al., 2015), anti‐obesity (Cha et al., 2013; Choo & Shin, 1999; Kim & Lim, 2014; Koo, Seong, Kown, Sohn, & Cha, 2008; Lee, Cha, Park, & Lee, 2017; Shin et al., 2016; Son et al., 2018; Yang, Kim, Kim, Lee, & Hong, 2019), anti‐diabetic (Kang et al., 2011; Kwon et al., 2009; Yang et al., 2019), and anti‐cancer (Park, Kong, Jung, & Rhee, 2001) effects. The major ingredients of *gochujang* are red pepper, grains (such as white, brown, and glutinous rice, barley, and wheat), and *meju* (fermented soybean powder). Capsaicin and its analogs in red pepper are reported to increase energy consumption by β‐adrenergic stimulation (Choo & Shin, 1999; Kawada, Hagihara, & Iwai, 1986; Sun et al., 2018), regulate lipid levels in the blood (Negulesco, Young, & Ki, 1985, Negulesco et al., 1987), enhance the immune response (Reyes‐Escogido, Gonzalez‐Mondragon, & Vazquez‐Tzompantzi, 2011), and reduce inflammatory adipocytokine release from adipose tissue (Kang et al., 2010; Kang, Kim, Han, Kawasa, & Yu, 2007). Furthermore, previous studies have demonstrated that fermented soybean (*meju*) or soybean *koji* fermented with various generally recognized as safe filamentous fungi has anti‐obesogenic and anti‐atherogenic properties (Anderson, Johnstone, & Cook‐Newell, 1995; Velasquez & Bhathena, 2007) and antioxidative activity (Lin, Wei, & Chou, 2006).

Various secondary metabolites with biological properties are generated by enzymatic degradation of the raw ingredients of *gochujang* via microbes in the fermentation process (Jang et al., 2017; Lee, Lee, et al., 2016; Lee, Shin, et al., 2016; Lee et al., 2017; Shin et al., 2012). *Koji* used in *gochujang* production comprises cooked grains and/or soybeans that have been inoculated with a fermentation culture, the filamentous fungus *Aspergillus oryzae.* In addition to *Aspergillus*, the bacteria genus *Bacillus* is also widely used for fermenting soybean (Lee, Lee, et al., 2016). Recently, it was reported that the functional components of the secondary metabolites of *gochujang* fermented by *Aspergillus* spp. and *Bacillus* spp. (Lee, Lee, et al., 2016; Lee, Shin, et al., 2016; Lee et al., 2017) vary depending on the types of microorganisms or cereals used in *koji* production, and these components have various biological activities (Lee, Lee, et al., 2016; Lee, Shin, et al., 2016; Shin et al., 2012, 2016). Amino acid and citric acid contents were high in WG, whereas flavonoids, soyasaponins, and γ‐aminobutyric acid (GABA) contents were high in RG (Lee, Lee, et al., 2016). The antioxidative effect of WG was higher than that of RG (Lee, Shin, et al., 2016). However, no studies have been conducted to compare the anti‐obesity effects of RG and WG in diet‐induced obese rats.

Several studies have also found that bioactive compounds showed different patterns and bioactive properties depending on cereal types (Lee, Lee, et al., 2016; Lee, Shin, et al., 2016; Lee, Suh, Jung, & Lee, 2016; Shin et al., 2012). They used giant embryonic rice (GE rice), a mutant rice, YR233517Acp79, with an enlarged embryo and contains higher γ‐oryzanol, tocopherol, GABA, protein vitamins, essential amino acids, and minerals compared with normal embryonic rice (NE rice) (Chung, Lee, & Kang, 2017; Chung, Lo, Nam, Jin, & Kang, 2016; Seo et al., 2011). The GE rice exhibited higher body fat‐lowering and hypolipidemic effects than the NE rice (Chung, Kim, Rico, & Kang, 2014).

In this study, the secondary metabolites of RG and WG prepared using GE rice *koji* and wheat *koji* were measured, and the inhibitory effects of *gochujang* prepared with various *koji* types on obesogenic and hyperlipidemic properties in high‐fat diet (HFD)‐induced obese rats were investigated.

## 2 MATERIALS AND METHODS

### 2.1 Preparation of *gochujang*

The g*ochujang* samples were provided by the CJ Cheiljedang Corporation. Commercial *gochujang* was made through a modified processing method. The two types of *gochujang* were distinguished by the method in which the *koji* was processed. GE rice *koji* was cultured at 37°C for 30 hr by inoculating the starter strain (*Aspergillus oryzae* CJ 1354) into steamed GE rice for RG. The wheat *koji* was cultured at 35°C for 30 min by inoculating the starter strain (*Aspergillus oryzae* CJ KY) into steamed wheat flour for WG. For the production of soybean *meju*, a bacterium (*Bacillus amyloliquefaciens* CJ 14‐6) was cultured in soy sauce BASE medium at 37°C and 200 rpm for 24 hr, and 2% of the bacteria were inoculated to the soybean supplemented with the culture broth against the original soybean. The inoculated soybeans were cultured at 37°C for 36 hr, while controlling the temperature of the product. The emulsified soybean *meju* was dried at 35°C for 24 hr, pulverized, and used as *meju* powder. *Gochujang* was produced by mixing GE rice or wheat *koji* prepared, wheat gluten, *meju* powder (modified *meju*), and cultured yeast broth, followed by maturation at 30°C for 20 days. Finally, for the production of *gochujang*, the matured materials (rice or wheat semifinished product), red pepper powder, red pepper seasoning, syrup, refined salt, and purified water were mixed and chopped after sterilization for 70 min at 70°C. The g*ochujang* samples were freeze‐dried and ground into powder using a grinder machine (HMF‐3250S, Hanil Electronics), and were stored at −70°C.

### 2.2 Analysis of nutrient components, amino acids and fatty acids composition

Moisture, protein, lipid, and ash contents of *gochujang* samples were measured according to the method of the Association of Official Analytical Chemists (AOAC) (2005). Dietary fiber was determined by the slightly modified AOAC enzymatic–gravimetric method (Mongeau & Brassard, 1993), and calories were analyzed using a calorimeter (PARR 1351 Bomb Caloriemeter). Amino acid content was determined using the hydrolysis/high‐performance liquid chromatography (HPLC) method (Henderson, Ricker, Bidlingmeyer, & Woodward, 2000). The fatty acid compositions were measured based on a well‐established method (Folch, Lees, & Sloane‐Stanley, 1957; Lepage & Roy, 1986). Each test was replicated three times for each experimental group.

### 2.3 Sample extraction and metabolite profiling analysis of *gochujang*

Each *gochujang* sample (500 mg) was extracted with 1 ml of 80% methanol, using a Mixer Mill (Grindomix GM 200; Retsch GmbH & Co.) at 30 Hz/s for 10 min and then centrifuged at 14,336 *g* for 10 min at 4°C. The supernatant was concentrated and dried in a speed vacuum concentrator (Biotron). The extracted sample was re‐dissolved in the same solvent and filtered through a 0.2 μm polytetrafluoroethylene membrane. Phenolic compounds in samples were analyzed by ultrahigh‐performance liquid chromatography and mass spectrometry (UPLC‐Q‐TOF‐MS), using a UPLC system (Waters Corp.) combined with a Q‐TOF Premier MS (Waters Micromass Technologies) system, coupled with an ultraviolet (UV) detector and an autosampler. The *gochujang* sample extract (5 ml) was injected into an ACQUITY BEH C18 Column (100 × 2.1 mm i.d., 1.7 μm; Waters Corp.). The gradient mobile phase consisted of solvent A (water) and solvent B (acetonitrile) with 0.1% formic acid. The mobile phase gradient was initially maintained at 5% B for 1 min, raised to 100% B over 10 min at a flow rate of 0.3 ml/min, held at 100% B for 2 min, and then finally decreased to 5% B over 1 min. The metabolites were analyzed in both positive and negative ion modes under the following operating conditions: cone voltage, 40 V; capillary voltage, 2.5 kV; mass range, m/z 100–1,000; and source and desolvation temperatures of 100°C and 300°C, respectively. Capillary voltages were set at −2.3 kV and +2.5 kV in negative and positive ionization modes, respectively. All peak data were analyzed using Mass Lynx software (version 4.1; Waters Corp.).

### 2.4 Animals and diets

Forty male, 5‐week‐old Sprague Dawley rats were purchased from the Orient Bio Co., Ltd. and used in the experiment after they were assimilated on a commercial diet for 1 week. The rats were assigned to groups according to body weight with ten rats per group, and they were separated from each another, with one rat per stainless steel cage, and bred for 8 weeks. The rats were divided into four groups: normal diet (ND), high‐fat diet (HFD), high‐fat diet with 10% GE rice *koji*‐supplemented *gochujang* (HFD‐RG), and high‐fat diet with 10% wheat *koji*‐supplemented *gochujang* (HFD‐WG). Experimental diets were prepared by modification of the AIN‐93 purified diet (Reeves, Nielson, & Fahey, 1993), as described in Table 1. The experimental animal breeding room was maintained at 22 ± 1°C and the relative humidity was maintained at 65 ± 5%. The lighting was adjusted at 12‐hr intervals (09:00 to 21:00). Water and food were provided ad libitum throughout the experimental period. Body weight and food intake were measured once a week at a specific time point.

**Table 1:** Composition of experimental diet

Diet composition (g/kg diet)	Diet groups			
	ND	HFD	HFD‐RG	HFD‐WG
Casein	200.0	200.0	200.0	200.0
L‐cystine	3.0	3.0	3.0	3.0
Corn starch	417.986	217.6	117.6	117.6
Sucrose	100.0	100.0	100.0	100.0
Dextrose	132.0	132.0	132.0	132.0
Cellulose	50.0	50.0	50.0	50.0
Lard		200.0	200.0	200.0
Soybean oil	50.0	50.0	50.0	50.0
Mineral mix^1^	35.0	35.0	35.0	35.0
Vitamin mix^1^	10.0	10.0	10.0	10.0
Choline chloride	2.0	2.0	2.0	2.0
t‐Butylhydroquinone	0.014	0.4	0.4	0.4
Rice *koji*‐added *gochujang* powder			100.0	
Wheat *koji*‐added *gochujang* powder				100.0

Abbreviations: HFD, high‐fat diet; HFD‐RG, high‐fat diet with 10% giant embryonic rice *koji*‐supplemented *gochujang*; HFD‐WG, high‐fat diet with 10% wheat *koji*‐supplemented *gochujang*; ND, normal diet.

^1^ AIN‐93‐MX mineral mixture and AIN‐93‐VX vitamin mixture (Reeves et al., 1993).

### 2.5 Serum and tissue sampling procedure

Experimental animals were fasted for 12 hr after the end of the test period, and body weights were measured immediately before the autopsy was performed. Blood was collected from the abdominal aorta after light anesthesia with CO_2_, and serum was separated by centrifugation at 1,088 *g* for 20 min at 4°C. Liver and white fat pads (i.e., epididymal, mesenteric, retroperitoneal, and perirenal fat pads) were weighed and the relative weight per 100 g of fasted body weight was calculated. All samples were stored at −70°C for lipid profile analysis.

### 2.6 Measurement of serum lipid profiles, leptin content, and enzyme activity

Serum TG, total cholesterol (TC), and HDL‐cholesterol (HDL‐C) levels, as well as activities of alanine transaminase (ALT), aspartate transaminase (AST), alkaline phosphatase (ALP), and lactate dehydrogenase (LDH) were measured using a blood biochemistry analyzer (Fugi Dri‐Chem 3,500; Fujifilm). LDL/VLDL‐C content was measured using an enzyme assay kit (BioVision Inc.). Serum leptin levels were measured using an ELISA kit (R&D Systems).

### 2.7 Measurement of TG and TC contents in tissues

Total lipid was extracted following the methods of Folch et al. (1957) for the analysis of TG and TC contents in the liver and epididymal adipose tissue. Chloroform‐methanol (2:1 v/v) was added to 0.1 g of the liver and adipose tissue, and samples were maintained for 3 days in a refrigerated state. Distilled water was added, followed by centrifugation at 3,000 rpm for 20 min, and the lower layer of the lipid was used. TC and TG contents were measured following the methods of Zlatkis and Zak (1969) and Biggs, Erikson, and Moorehead (1975), respectively.

### 2.8 Hepatic lipid accumulation

A portion of extracted liver tissues was collected immediately after the dissection of the rats. After fixation of the tissues in 4% paraformaldehyde solution, tissue sections (3–4 μm thick) were prepared using the Cryocut Microtome (CM1800, Leica) at −25°C, attached to a slide, and dried. The slides were stained with Oil‐Red O, followed by rinsing, neutralization, and dehydration. They were observed under a light microscope following encapsulation with an encapsulating agent.

### 2.9 Adipocyte size

Epididymal adipose tissues were extracted and fixed in 10% neutral formalin solution. Next, the tissue was embedded in paraffin, and slides were prepared and stained with hematoxylin‐eosin (H & E). To measure epididymal adipocyte size, the slides were observed using an electron microscope at a magnification of 100×. The adipocyte sizes of the treated sections were compared using ImageJ software (National Institute of Mental Health).

### 2.10 Lipid‐related enzyme activities in liver and lipoprotein lipase (LPL) activity in adipose tissue

The activities of fatty acid synthase (FAS), acetyl CoA carboxylase (ACC), glucose‐6‐phosphate‐dehydrogenase (G6PDH), and malic enzyme (ME) were determined in liver. Hepatic tissues were rinsed three times with 0.1 M potassium phosphate buffer (pH 7.40) at 37°C and then homogenized. The homogenate was centrifuged at 3,000 rpm for 15 min (Combi‐514R, Hanil), and the top layer was collected and used to measure the enzyme activity. The enzyme activities of FAS, ACC, G6PDH, and ME were measured using spectrophotometric methods according to the procedures described by Linn (1981), Zabala et al. (2004), Lohr and Waller (1974), and Kouba and Mourot (1998), respectively. Enzyme activity was expressed as nmol nicotinamide adenine dinucleotide phosphate per min per gram of tissue. Protein contents were assayed with a commercial kit (BCA Protein Assay, Thermo Fischer Scientific). Heparin‐releasable lipoprotein (HR‐LPL) and total extractable LPL (TE‐LPL) activities were assayed as described by Fried, Velazquez, and Nobel (1990) and Iverius and Brunzell (1985), respectively. HR‐ and TE‐LPL activities in the heparin elute were measured using the glycerol stabilized ^3^H‐triolein emulsion as substrate. One unit of LPL activity was defined as the release of 1 μmol of free fatty acid in 1 hr.

### 2.11 Statistical analysis

Experimental results were analyzed using SPSS version 21.0 (SPSS Inc.). The results are presented as means ± standard error for each group. Student's *t* tests were performed to assess the statistical significance of differences between two groups. One‐way analysis of variance was conducted to evaluate differences in means among three or more groups, and significant differences were examined by post hoc Tukey's tests at *p* < .05.

## 3 RESULTS

### 3.1 Nutrient components and metabolite profiling of *gochujang* samples

The results showing proximate constituents, free amino acid and fatty acid compositions, and secondary metabolites of *gochujang* prepared using GE rice or wheat *koji* are listed in Tables 2 and 3, respectively. Significantly different metabolites, including amino acids, fatty acids, flavonoids, soyasaponins, alkaloids, and lipids, were identified according to the type of grains used in *gochujang*. Fat, moisture, ash, and dietary fiber contents were higher in RG than in WG. However, the carbohydrate, protein, and calories were higher in WG than in RG. The contents of most essential amino acids (threonine, valine, methionine, isoleucine, leucine, phenylalanine, and lysine) were significantly higher in WG than in RG. The contents of total amino acids and essential amino acids were significantly higher in WG compared with RG. Oleic acid and linolenic acid contents were high in RG, whereas linoleic acid content was higher in WG than in RG.

**Table 2:** Nutrient components of *gochujang* prepared with rice and wheat *koji*

	Rice *koji*‐added *gochujang*	Wheat *koji*‐added *gochujang*
Proximate composition		
Moisture (%)	8.21 ± 0.42^1^	7.43 ± 0.31
Carbohydrate (%)	68.42 ± 3.64	70.29 ± 4.10
Protein (%)	8.37 ± 0.34*	9.82 ± 0.59
Fat (%)	1.10 ± 0.08***	0.61 ± 0.02
Ash (%)	14.12 ± 0.84**	12.14 ± 0.77
Dietary fiber (%)	10.21 ± 0.22***	8.01 ± 0.32
Energy (kcal/100 g)	316.34 ± 9.21	309.27 ± 4.51
Free amino acid composition		
Aspartic aid	0.42 ± 0.02	ND
Threonine	10.10 ± 0.36**	14.77 ± 0.29
Serine	7.48 ± 0.28***	13.66 ± 0.47
Asparagine	2.33 ± 0.01***	6.27 ± 0.12
Glutamic acid	6.40 ± 0.09**	8.84 ± 0.14
α‐Aminoadipic acid	0.89 ± 0.03***	0.32 ± 0.02
Proline	35.86 ± 0.59***	68.81 ± 0.97
Glycine	4.59 ± 0.16**	6.83 ± 0.33
Alanine	19.93 ± 0.51**	28.78 ± 0.47
Citrulline	1.57 ± 0.05	ND
Valine	25.46 ± 0.63***	41.62 ± 0.54
Cystine	ND	0.52 ± 0.03
Methionine	6.60 ± 0.08***	15.18 ± 0.09
Isoleucine	22.88 ± 0.34***	37.81 ± 0.67
Leucine	38.09 ± 0.70***	74.62 ± 0.66
Tyrosine	15.84 ± 0.31**	30.49 ± 0.39
Phenylalanine	22.93 ± 0.64***	49.32 ± 0.50
β‐Alanine	ND	0.48 ± 0.03
β‐Aminoisobutyric acid	ND	2.69 ± 0.09
γ‐Amino‐*n*‐butyric acid	6.12 ± 0.41_*_	7.07 ± 0.28
Histidine	1.42 ± 0.03**	2.48 ± 0.04
Carnosine	4.51 ± 0.18	ND
Lysine	ND	4.21 ± 0.08
Fatty acid composition		
Luric acid (C12:0)	0.40 ± 0.06*	0.22 ± 0.04
Myristic acid (C14:0)	0.40 ± 0.02***	2.34 ± 0.05
Palmitic acid (C16:0)	20.74 ± 0.57	22.05 ± 0.85
Heptadecanoic acid (C17:0)	1.72 ± 0.08*	1.50 ± 0.06
Stearic acid (C18:0)	6.48 ± 0.05**	3.53 ± 0.44
Arachidic acid (C20:0)	0.69 ± 0.67***	0.37 ± 0.64
Heneicosanoic acid (C21:0)	ND	10.37 ± 0.25
Behenic acid (C22:0)	0.68 ± 0.13**	0.38 ± 0.24
Lignoceric acid (C24:0)	0.36 ± 0.07	0.34 ± 0.14
Saturated	32.73	39.77
Palmitoleic acid (C16:1)	0.96 ± 0.07**	0.49 ± 0.14
Oleic acid (C18:1n9c)	20.96 ± 0.57**	14.01 ± 0.37
Monounsaturated	22.24	15.01
Linoleic acid (C18:2n6c)	36.01 ± 0.64*	40.11 ± 0.43
Linolenic acid (C18:3n3)	8.81 ± 0.64***	5.11 ± 0.64
cis−8,11,14‐Eicosatrienoic acid (C20:3n6)	0.14 ± 0.13	ND
Arachidonic acid (C20:6n3)	0.08 ± 0.13	ND
Polyunsaturated	45.04	45.22

Note

Significant difference between rice *koji* and wheat *koji* at *p < .05, **p < .01, ***p < .001 by Student's *t* test.

^1^ Values are mean ± *SD* (*n* = 6).

**Table 3:** Relative contents of targeted secondary metabolites in *gochujang* products

Tentative matabolite	Contents (peak area)	
	Rice *koji‐*added *gochujang*	Wheat *koji‐*added *gochujang*
*Flavonoids*		
Luteoline‐diglucosdie	2.7 ± 0.3^1^	3.4 ± 0.3
Genistin	4.4 ± 0.3	2.8 ± 0.9
Apigenin‐diglucoside	15.2 ± 0.3	12.0 ± 0.7
Daidzein	105.0 ± 2.6*	10.7 ± 0.7
Glycitein	26.2 ± 0.8*	1.8 ± 0.7
Genistein	121.2 ± 4.4*	19.1 ± 1.9
Kaempferol	2.3 ± 0.2	3.8 ± 0.3
*Soyasaponins*		
Soyasaponin V	8.5 ± 0.5	1.3 ± 0.6
Soyasaponin I	98.2 ± 3.5*	9.8 ± 0.7
Soyasaponin II	4.3 ± 0.2*	0.4 ± 0.1
Soyasaponin III	12.1 ± 0.6	1.3 ± 0.2
*Alkaloids*		
Capsaicin	16.6 ± 0.9*	22.1 ± 1.3
Dihydrocapsaicin	8.3 ± 0.5*	12.2 ± 1.2
*Lipids*		
LysoPC16:0	26.9 ± 14.5	31.3 ± 1.8
LysoPC18:1	13.9 ± 4.4	10.0 ± 0.3
LysoPC18:2	14.0 ± 8.8	20.5 ± 1.5

Note

Values with different superscripts in the same row are significantly different (*p*<0.05) between groups by Tukey's test

^1^ Values are mean ± *SD* (*n* = 6).

The contents of isoflavone compounds such as daidzein, glycitein, and genistein, and saponin contents such as soyasaponin I and II, were ten times higher in RG than in WG. In contrast, the capsaicin and dihydrocapsaicin contents were significantly higher in WG than in RG. However, there were no significant differences in lysophosphatidylcholines (lysoPC) content and composition of RG and WG.

### 3.2 Body weight, food intake, food efficiency ratio (FER), and liver and white fat pad weights

Table 4 shows the body weight gain, food intake, and FER for rats fed with an HFD or a diet supplemented with different *gochujang* types. Body weight gain and FER were significantly higher in the HFD groups (HFD, HFD‐RG, and HFD‐WG) than in the ND group. However, body weight gain in the *gochujang*‐supplemented groups was significantly lower than in the HFD group. Body weight gain of rats in the HFD‐RG group tended to be lower than that of the HFD‐WG group. Food intake in the HFD groups was significantly lower than in the ND group. FER showed a significant decrease in the *gochujang*‐supplemented groups than in the HFD group, but there was no significant difference between the *gochujang* groups.

**Table 4:** Changes in body weight gain, food intake, food efficiency ratio, and the relative liver and adipose tissue weights of rats fed experimental diets

	ND	HFD	HFD‐RG	HFD‐WG
Initial body weight (g)	195.44 ± 10.62^1^	197.13 ± 11.29	197.25 ± 9.87	197.81 ± 11.01
Final body weight (g)	532.88 ± 21.32^c^	613.13 ± 43.21^a^	563.19 ± 35.20^b^	577.81 ± 39.21^b^
Body weight gain (g/day)	6.03 ± 0.07^c^	7.45 ± 0.05^a^	6.54 ± 0.10^b^	6.79 ± 0.07^b^
Food intake (g/day)	18.52 ± 0.19^a^	16.61 ± 0.23^b^	16.74 ± 0.20^b^	16.91 ± 0.30^b^
FER (%)^2^	0.33 ± 0.00^c^	0.33 ± 0.00^c^	0.40 ± 0.00^b^	0.40 ± 0.01^b^
Relative liver weight (g/100 g body wt.)	2.39 ± 0.01^b^	2.85 ± 0.09^a^	2.46 ± 0.06^b^	2.50 ± 0.04^b^
Relative adipose fat weight (g/100 g body wt.)				
Epididymal fat	1.82 ± 0.06^c^	2.41 ± 0.05^a^	2.07 ± 0.04^b^	2.14 ± 0.05^b^
Mesenteric fat	0.86 ± 0.04^b^	1.39 ± 0.04^a^	1.07 ± 0.02^ab^	1.10 ± 0.03^ab^
Retroperitoneal fat	2.03 ± 0.08^c^	2.83 ± 0.08^a^	2.28 ± 0.02^b^	2.25 ± 0.06^bc^
Perirenal fat	0.60 ± 0.04^b^	0.88 ± 0.04^a^	0.70 ± 0.03^b^	0.69 ± 0.02^b^
Total fat	5.31 ± 0.27^c^	7.51 ± 0.31^a^	6.12 ± 0.10^b^	6.18 ± 0.12^b^

Note

Values with different superscripts in the same row are significantly different (*p* < .05) between groups by Tukey's test.

Abbreviations: HFD, high‐fat diet; HFD‐RG, high‐fat diet with 10% giant embryonic rice *koji*‐supplemented *gochujang*; HFD‐WG, high‐fat diet with 10% wheat *koji*‐supplemented *gochujang*; ND, normal diet.

^1^ Values are mean ± *SE* (*n* = 10).

^2^ FER (food efficiency ratio): weight gain (g/day)/ food intake (g/day).

The weights of liver and white fat pads were the relative weights per 100 g of fasted body weight calculated (Table 4). Weights of liver and total white fat pads were significantly higher in the HFD groups than in the ND group. The liver weights of rats in the *gochujang*‐supplemented groups were significantly lower than those of the rats in the HFD group, but were not significantly different from those of the rats in the ND group. Weights of epididymal, retroperitoneal, mesenteric, and perirenal fat pads were significantly lower in the *gochujang*‐supplemented groups than in the HFD group, but there were no significant differences between the *gochujang* groups.

### 3.3 Serum lipid profiles, and ALT, AST, ALP, and LDH activities

Serum TG contents were significantly lower in the *gochujang*‐supplemented groups than in the HFD group (Table 5). TC contents of the HFD‐RG group were significantly lower than those of the HFD group. LDL/VLDL‐C contents were significantly higher in the HFD groups than in the ND group, but they were significantly lower in the *gochujang*‐supplemented groups than in the HFD group. HDL‐C contents were significantly lower in the HFD groups than in the ND group, and the *gochujang*‐supplemented groups tended to have increased HDL‐C compared to the HFD group. However, there was no significant difference between the two *gochujang* groups.

**Table 5:** Serum lipid profiles, and ALT, AST, ALP, and LDH activities in rats fed experimental diets

	ND	HFD	HFD‐RG	HFD‐WG
Lipid profiles (mg/dL)				
Triglyceride	61.38 ± 5.50^1^ ^,c^	97.25 ± 9.03^a^	79.13 ± 4.66^b^	82.50 ± 6.77^b^
Total cholesterol	103.88 ± 8.17^b^	141.13 ± 7.94^a^	112.75 ± 8.01^b^	112.75 ± 8.01^b^
LDL/VLDL cholesterol	41.23 ± 5.01^c^	88.23 ± 5.98^a^	69.38 ± 4.98^b^	79.05 ± 6.21^ab^
HDL‐cholesterol	49.31 ± 3.29^a^	31.29 ± 2.43^b^	38.49 ± 4.19^b^	37.33 ± 3.89^b^
Serum cardiac biomarkers (U/L)				
ALT	31.38 ± 2.54^b^	54.00 ± 3.87^a^	45.25 ± 3.36^a^	46.75 ± 3.35^a^
AST	94.63 ± 4.80^b^	120.38 ± 5.21^a^	102.13 ± 6.40^ab^	110.00 ± 5.56^ab^
ALP	126.25 ± 3.16^NS^	138.13 ± 5.98	130.50 ± 6.85	133.25 ± 6.62
LDH	438.63 ± 11.43^b^	567.88 ± 17.75^a^	434.25 ± 28.84^b^	493.63 ± 31.74^ab^

Note

Values with different superscripts in the same row are significantly different (*p* < .05) between groups by Tukey's test.

Abbreviations: HFD, high‐fat diet; HFD‐RG, high‐fat diet with 10% giant embryonic rice *koji*‐supplemented *gochujang*; HFD‐WG, high‐fat diet with 10% wheat *koji*‐supplemented *gochujang*; ND, normal diet.

^1^ Values are mean ± *SE* (*n* = 10).

Serum ALT, AST, and LDH activities (Table 5) were significantly higher in the HFD group than in the ND group. The *gochujang*‐supplemented groups tended to have lower ALT, AST, and LDH activities than the HFD group, but there was no significant difference between the two *gochujang* groups. Furthermore, there were no significant differences in serum ALP activity among the experimental groups.

### 3.4 TG and TC contents and histopathological changes in the liver

**Figure 1:**
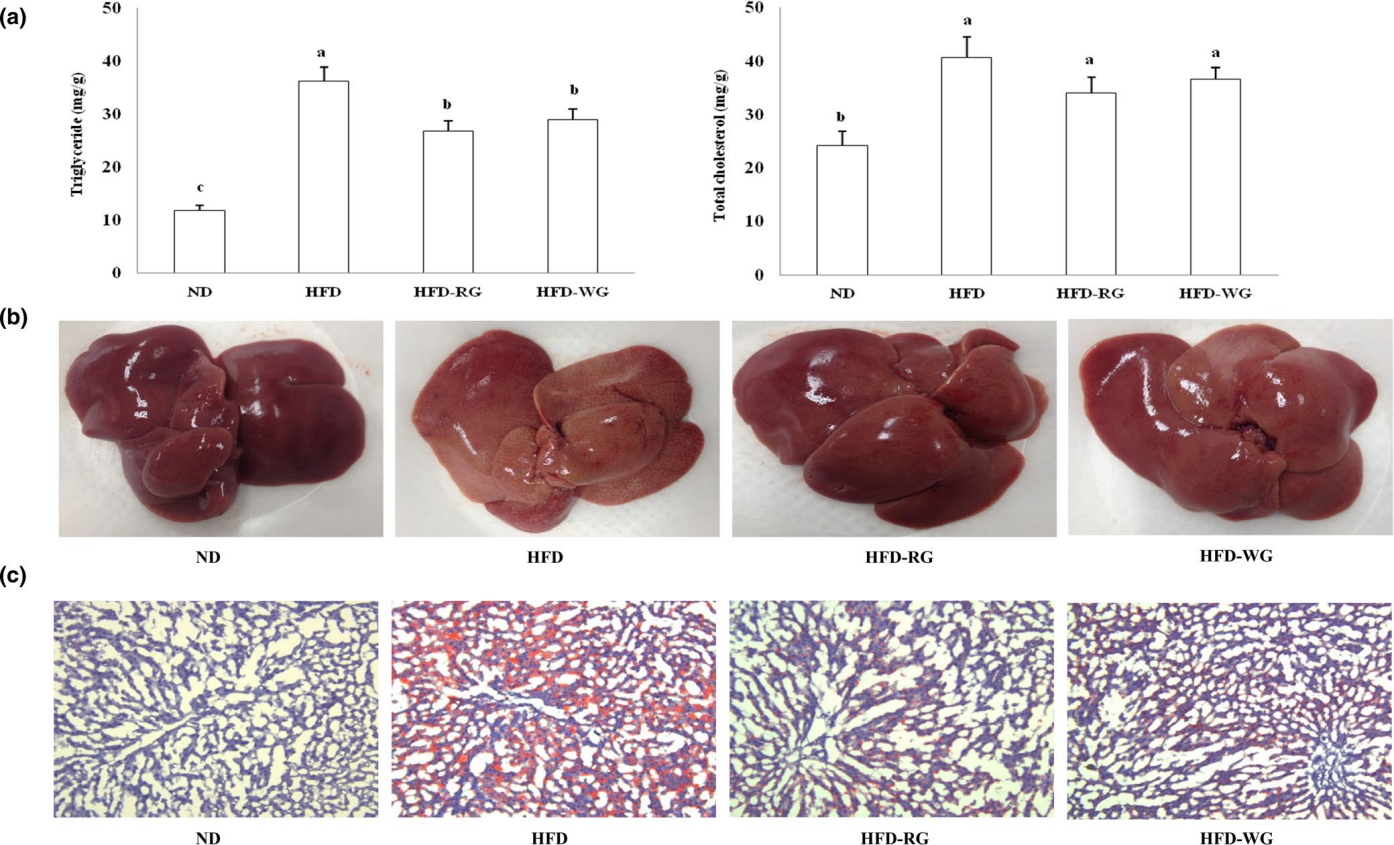
(a) Triglyceride and total cholesterol levels, (b) representative anatomical views, and (c) histopathological analysis of the livers of rats fed experimental diets for 8 weeks. ND: normal diet; HFD: high‐fat diet; HFD‐RG: high‐fat diet with 10% giant embryonic rice *koji*‐supplemented *gochujang*; HFD‐WG: high‐fat diet with 10% wheat *koji*‐supplemented *gochujang*. Values are means ± *SE* (*n* = 10). Bars with different letters are significantly different at *p* < .05 by Tukey's tests

The shape of the liver and the Oil‐Red O staining were examined to determine the lipid contents and lipid accumulation in the liver, and the results are summarized in Figure 1a–c. The TG and TC contents in the liver were higher in the HFD group than in the ND group. The hepatic TG content was significantly lower in the HFD‐RG group than in the HFD group. However, TC content in liver showed no difference between all *gochujang* groups and HFD group (Figure 1a). The shape of the liver (Figure 1b) photographed immediately after the sacrifice of the rats, was dark red in the ND group, but light pink with yellow fat deposits in the HFD group. The *gochujang*‐supplemented groups showed less fat deposition than the HFD group. In addition, when the liver tissues stained with Oil‐Red O were observed under an optical microscope (Figure 1c), they were more reddish in color. In addition, fat deposition was clearly observed in the HFD group compared to the ND group. However, the *gochujang*‐supplemented groups showed reductions in number and size of lipid droplets compared to the HFD group. Steatosis was observed in the HFD groups of rats (HFD, HFD‐RG, and HFD‐WG groups).

### 3.5 Leptin content in serum, TG and TC contents in epididymal adipose tissue, and the size of epididymal adipocytes

**Figure 2:**
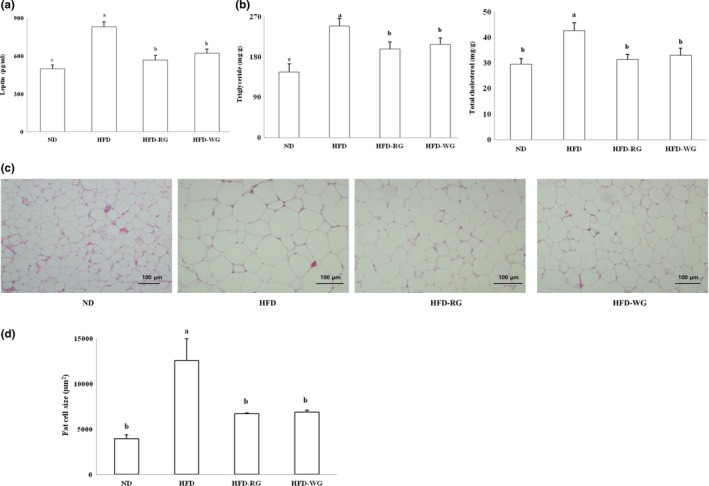
(a) Leptin levels in serum, (b) triglyceride and total cholesterol levels, (c) representative findings, and (d) adipocyte sizes of epididymal adipose tissue. ND: normal diet; HFD: high‐fat diet; HFD‐RG: high‐fat diet with 10% giant embryonic rice *koji*‐supplemented *gochujang*; HFD‐WG: high‐fat diet with 10% wheat *koji*‐supplemented *gochujang*. Values are means ± *SE* (*n* = 10). Bars with different letters are significantly different at *p* < .05 by Tukey's tests. Bar = 100 μm

Serum leptin content (Figure 2a) was significantly higher in the HFD groups than in the ND group, and the *gochujang*‐supplemented groups showed significant decreases compared to the HFD group. The change in the lipid profile of epididymal adipose tissues (Figure 2b) indicated significant decreases in TG and TC contents in the *gochujang*‐supplemented groups compared to those in the HFD group. The size of lipid droplets that had accumulated in the epididymal adipocytes was observed with a microscope after H&E staining of the samples (Figure 2c). The results obtained using an Image Analyzer (Figure 2d) demonstrated that adipocytes sizes were significantly larger in the HFD group than in the ND group. However, the adipocytes sizes were significantly smaller in the *gochujang*‐supplemented diet groups than in the HFD group.

### 3.6 Changes in the activities of metabolic enzymes related to lipid synthesis

The activity of enzymes related to lipid synthesis in the liver and epididymal adipose tissue were investigated, as shown in Table 6. In the liver, the activities of FAS, ACC, and ME were significantly elevated in the HFD group compared to the ND group. The activities of FAS, ACC, and ME were significantly lower in *gochujang*‐supplemented diet groups than in the HFD group, with higher inhibition in the HFD‐RG group than in the HFD‐WG group. *Gochujang* treatment showed a slight inhibition in the hepatic enzyme related‐lipid synthesis activity, although this was not statistically significant. In epididymal adipose tissue, HR‐LPL and TE‐LPL activities were also significantly elevated in the HFD group compared to the ND group, but inhibited in the *gochujang*‐supplemented groups. There was no significant difference between the two *gochujang* groups.

**Table 6:** Effects of *gochujang* supplementation on FAS, ACC, G6PDH, and ME activities in liver, and HR‐LPL and TE‐LPL activities in epididymal adipose tissue

	ND	HFD	HFD‐RG	HFD‐WG
Liver (nmol/min/protein)				
FAS	10.29 ± 0.69^c^	16.32 ± 0.72^a^	13.02 ± 0.93^b^	13.13 ± 0.65^b^
ACC	0.16 ± 0.02^c^	0.45 ± 0.03^a^	0.29 ± 0.03^b^	0.30 ± 0.02^b^
G6PDH	20.03 ± 1.87^NS^	25.84 ± 1.93	22.32 ± 1.66	23.21 ± 0.99
ME	85.33 ± 8.12^c^	132.69 ± 10.36^a^	104.33 ± 7.68^b^	109.06 ± 6.95^b^
Epididymal adipose tissue (U/g)				
HR‐LPL	5.53 ± 0.98^b^	8.14 ± 0.57^a^	6.09 ± 0.54^b^	6.22 ± 0.6^b^
TE‐LPL	15.43 ± 1.42^c^	34.26 ± 2.34 ^a^	25.32 ± 1.96^b^	29.13 ± 2.17^ab^

Note

Values with different superscripts in the same row are significantly different (*p* < .05) between groups by Tukey's test.

Abbreviations: HFD, high‐fat diet; HFD‐RG, high‐fat diet with 10% giant embryonic rice *koji*‐supplemented *gochujang*; HFD‐WG, high‐fat diet with 10% wheat *koji*‐supplemented *gochujang*; ND: normal diet.

^1^ Values are mean ± *SE* (*n* = 10).

## 4 DISCUSSION

Functional components of *gochujang*, a traditional Korean hot sauce, have been identified as capsaicin (Kawada et al., 1986; Maclean, 1985; Negulesco et al., 1985), cooked grains or grains *koji* (Lee, Shin, et al., 2016), and soybean fermentation products (Anderson et al., 1995; Velasquez & Bhathena, 2007). Many recent studies of the fermented metabolites related to *gochujang* or its ingredients have examined fermentation products using various types of cereals, soybeans, and microorganisms (Jang et al., 2017; Lee, Lee, et al., 2016; Lee et al., 2017; Shin et al., 2012). Moon and Kim (1988) examined the composition percentage of raw materials used in *gochujang* prepared using rice and wheat, and they reported that the protein content in wheat was higher than in rice. The wheat *koji* was shown to have greater protease activity than glutinous rice *koji* (Park & Park, 1979). Many studies have also examined the metabolites and functionality of *gochujang* produced using different *koji* types (Lee, Shin, et al., 2016; Lee, Suh, et al., 2016). *Gochujang* product prepared using wheat *koji* has high contents of amino acids and sugar, whereas *gochujang* product prepared using rice *koji* has high contents of flavonoids, LysoPCs, and GABA (Lee, Shin, et al., 2016). The antioxidative activity, as well as flavonoids and LysoPCs contents in the rice *koji* produced by fermentation with *Bacillus amyloliquefaciens* were significantly higher than those in the rice *koji* produced by fermentation with *Aspergillus oryzae* (Lee, Lee, et al., 2016). In this study, characteristic metabolites such as amino acids and fatty acids also revealed significant patterns depending on the grain type. In addition, isoflavone and soyasaponin compounds were more abundant in RG than in WG, whereas capsaicin and dihydrocapsaicin contents were greater in WG due to differences in soybean *koji* and red pepper powder contents. Therefore, these results suggest that starch materials, including rice or wheat, and additional ingredients, such as soybean *koji* and hot red pepper, contributed to the secondary metabolite compositions. Furthermore, these entities are involved in the modulation of obesity and hyperlipidemia (Daveby, Åman, Betz, & Musser, 1998; Hwang et al., 2005; Kim et al., 2009; Naaz et al., 2003; Oakenfull & Sidhu, 1990).

With respect to the functionality of *gochujang*, various studies have examined its anti‐obesity effect (Kim & Lim, 2014; Koo et al., 2008; Shin et al., 2016; Son et al., 2018). Fermented *gochujang* is reported to have greater anti‐obesity effects than nonfermented *gochujang* (Rhee, Kong, Jung, & Park, 2003; Shin et al., 2016). Furthermore, *gochujang* prepared by mixing the *koji* fermented with fungus and bacterial fermentation soybean *meju* was superior with respect to weight loss and improvements in serum lipid metabolism (Shin et al., 2016). We used an HFD rat model to evaluate and compare the anti‐obesity and hypolipidemia effects of two types of *gochujang*: RG and WG. The results of this study confirmed that obesity was induced in HFD rats, as evidenced by the weight gain in rats fed an HFD for 8 weeks. The body weight gain was significantly lower in the *gochujang*‐supplemented groups than in the HFD group, and there were no significant differences in food intake. Our results indicate that *gochujang* intake has anti‐obesity properties that inhibit body weight gain, without affecting food intake. Obesity develops when energy intake exceeds energy expenditure (Kopelman, 2000). FER was significantly lower in the *gochujang‐*supplemented groups than in the HFD group. These results suggest that *gochujang* has an ameliorative effect on HFD‐induced body weight gain by increasing energy expenditure, suppressing lipid production or inhibiting the absorption of excess food in diet. Choo (2000) showed that an HFD supplemented with the traditional *gochujang* results in a greater decrease in body weight than that of rats on HFD supplemented with red pepper powder. This result indicates that the weight loss was not induced only by red pepper powder. *Gochujang* prepared using rice or wheat *koji* in this study contained isoflavone compounds, such as genistein and daidzein, as well as glycitein and soyasaponins, which were derived from fermented soybean and are composites with various physiological activities (Daveby et al., 1998; Guo, Wu, Su, Yang, & Zhang, 2009; Hwang et al., 2005; Kim et al., 2009; Naaz et al., 2003; Oakenfull & Sidhu, 1990). Daidzein, an isoflavone present in soybeans, is shown to decrease the body weight gain in HFD‐induced obese mice (Hwang et al., 2005). Kim et al. (2009) reported that treatment with crude saponins isolated from soybean cake reduced the body weight gain in HFD‐induced obese rat. Therefore, the reduction in body weight gain induced by *gochujang* diet in rats is not only due to capsaicin contained in *gochujang*, but is likely also due to metabolites such as isoflavone and soyasaponin compounds, generated during the fermentation process.

Excess fat intake causes abnormalities in lipid metabolism, resulting in changes in blood and tissue lipid components, fat deposition in various tissues (such as the liver and blood vessels), and an increased risk of atherothrombosis (Wu, Kao, Wen, & Wu, 1993). To compare the effect of RG and WG on lipid profiles and lipid accumulation in serum, hepatic, and adipose tissue, the activity of enzymes related to lipogenesis were evaluated. *Gochujang* has been shown to have an effect on lipid metabolism in the current study. Thus, the serum levels of TG, TC, and LDL/VLDL‐C, as well as hepatic and adipose tissue levels of TG and TC, increased in response to HFD. However, the serum, hepatic, and adipose tissue lipid profiles were improved by treatment with *gochujang*. Moreover, we showed that treatment with *gochujang* prevented hepatic degenerative changes induced by HFD in obese rats. Activation of lipogenic‐related enzymes including FAS, ACC, G6PDH, and ME are highly related to elevated TG level. Thus, the ameliorating effect of *gochujang* on hepatic steatosis and TG level was shown to be related to the suppression of hepatic lipogenic‐related enzyme activity. Similarly, the lipogenic enzymes such as FAS, G6PDH, and ME were markedly inhibited by both the nonfermented *gochujang* mixture and the commercial *gochujang* products using different cereal and soybean *koji* (Shin et al., 2016). Koo et al. (2008) found that the serum and hepatic TG levels, as well as hepatic ACC mRNA gene expression were decreased in rats fed with fermented *gochujang* in addition to a HFD. Genistein and capsaicin have been reported to decrease serum TG and TC levels. They also inhibit the adipocyte differentiation process via activating AMP‐activated protein kinase and lipid accumulation in fat cells and experimental animals as well as reduce white fat pad mass; thus, genistein and capsaicin have anti‐obesity effects (Hwang et al., 2005; Naaz et al., 2003). Soyasaponin, an amphipathic oleanane triterpenoid glycoside with polar sugar chains in the nonpolar pentacyclic ring (Daveby et al., 1998), has also been shown to reduce serum and hepatic TG and TC levels and to inhibit hepatic FAS activity (Kim et al., 2009; Oakenfull & Sidhu, 1990). Therefore, among the secondary metabolites of RG and WG, genisteine, capsaicin, and soyasaponin seem to positively improve hyperlipidemia caused by an HFD.

The enzymes ALT and AST are present in the liver and heart muscle, and reflect liver hypertrophy and liver status. If the liver is damaged, the membrane permeability of hepatocytes is enhanced, resulting in increased AST and ALT levels in the blood; accordingly, the activity of these two enzymes can be used as indicators of liver damage (Plaa & Charbonneau, 1994; Sung, Lee, Shin, Chung, & Lim, 1998). Serum ALP activity is also increased by liver disease; LDH activity is known to be significantly elevated in acute hepatitis, early liver cancer, myocardial infarction, malignant anemia, and leukemia (Ki, Song, Ha, & Han, 1993). Increases in serum ALT, AST, and LDH activities in rats fed the HFD tended to decrease by supplementation with *Gochujang,* and all rats in these groups showed normal activity ranges. These results indicate that *gochujang* does not cause hepatocellular toxicity.

Leptin is a hormone produced by the obesity gene of adipocytes (Caro, Sinha, Kolaczynski, Zhang, & Considine, 1996). Leptin secretion is increased in obesity; thus, the serum leptin concentration is used as an indicator of body fat mass in obesity studies (Seo, Han, Park, Koh, & Lee, 2010). In this study, leptin level, visceral fat pads mass, and the size of epididymal adipocytes were significantly increased by a HFD, but they were decreased by the *gochujang* treatment. These results suggested that RG and WG decreased the body weight gain and body fat, and these effects may be related to the decrease in serum leptin levels and the inhibition of lipid accumulation in epididymal adipocytes. Adipose tissue plays an active role in regulating the metabolism of the whole body as a site of energy accumulation and storage. However, excessive accumulation of adipose tissue in the body is associated with an increased incidence of metabolic diseases, such as diabetes, hypertension, and hyperlipidemia (Lim, 2013). In the current study, the addition of *gochujang* has been shown to have an inhibitory effect on visceral fat pads weight induced by an HFD. Moreover, *gochujang* treatment decreased adipocytes size, caused at least in part by inhibiting effects on the lipogenic enzyme LPL activity. Several studies reported that the addition of *gochujang* to HFD results in significant decreases in fat pads weights compared to those of a HFD group (Koo et al., 2008; Rhee et al., 2003). One of the possible explanations for the decreased white adipose tissue weight, adipocyte size, and lipid accumulation may be explained by the effects of capsaicin, which exacerbates catecholamine secretion (Choo & Shin, 1999; Kawada et al., 1986), promoting the degradation of fat in white adipose tissue (Negulesco et al., 1987, 1985) and increasing heat production by increasing β‐adrenergic activity in brown adipose tissue, which is an energy‐consuming organ (Choo & Shin, 1999; Lee & Chyun, 2007; Sun et al., 2018). On the other hand, *gochujang* products including fermented soybean (*meju*) contained higher contents of isoflavone, soyasaponine, and LysoPCs compounds. Guo et al., (2009) reported that when daidzein‐derived substances were fed to HFD‐indeed obese mice, there were beneficial effects on body weight and adipose tissue weight. Saponin derived from soybean meal treatment significantly decreased the weights of the white fat pads and lipid droplet area of epididymal adipocytes in HFD‐induced obese mice (Kim et al., 2009). In the present study, the increases in white fat pads weight due to a HFD were decreased by feeding with *gochujang*, suggesting that *gochujang* may inhibit the activity of LPL.

## 5 CONCLUSION

Based on the above results, *gochujang* prepared using rice *koji* and wheat *koji* improves lipid profiles in the serum, liver, and adipose tissue and decreases body weight gain and the weights of the liver and white fat pads in rats fed a HFD. Moreover, it inhibited the formation of lipid droplets in the liver, lowered the activity of hepatic lipogenic‐related enzyme, and decreased the size of epididymal adipocytes. Anti‐obesity and lipid‐lowering properties of capsaicin, genistein, daidzein, soyasaponin, and lysoPCs are thought to contribute to obesity and lipid metabolism. RG, which has high contents of these components, with the exception of capsaicin, seems to be slightly more beneficial than WG with respect to anti‐obesity effect and lipid metabolism improvement. However, further studies are required to clarify detailed action mechanisms underlying the anti‐obesity effects of RG and WG.

## CONFLICT OF INTEREST

The authors declare no conflict of interest.

## ETHICAL APPROVAL

This study has conformed to the Declaration of Helsinki, US. All animal care and experimental protocols were ethically viewed and approved (Approval number: CIACUC2015‐A0028) by Institutional Animal Care and Use Committee of Chosun University, Korea.
